# Effect of Nano-SiO_2_ on the Early Hydration of Alite-Sulphoaluminate Cement

**DOI:** 10.3390/nano7050102

**Published:** 2017-05-03

**Authors:** Jinfeng Sun, Zhiqiang Xu, Weifeng Li, Xiaodong Shen

**Affiliations:** 1 College of Materials Science and Engineering, Nanjing Tech University, Nanjing 210009, China; jinfengsun1990@163.com (J.S.); aoju918@163.com (Z.X.); 2 State Key Laboratory of Materials-Oriented Chemical Engineering, Nanjing Tech University, Nanjing 210009, China

**Keywords:** nano-SiO_2_, AC$A cement, early hydration, properties

## Abstract

The impact of nano-SiO_2_ on the early hydration properties of alite-sulphoaluminate (AC$A) cement was investigated with a fixed water to solid ratio (*w*/*s*) of one. Nano-SiO_2_ was used in partial substitution of AC$A cement at zero, one and three wt %. Calorimetry, X-ray diffraction (XRD), thermogravimetric/derivative thermogravimetric (TG/DTG), mercury intrusion porosimetry (MIP) and scanning electron microscopy (SEM) analyses were used to characterize the hydration and hydrates of the blended cement. The hydration of the AC$A cement was significantly promoted, resulting in an increase of the heat released with the addition of nano-SiO_2_. Phase development composition analysis showed that nano-SiO_2_ had no effect on the type of crystalline hydration products of the AC$A cement. Moreover, nano-SiO_2_ showed significant positive effects on pore refinement where the total porosity decreased by 54.09% at three days with the inclusion of 3% nano-SiO_2_. Finally, from the SEM observations, nano-SiO_2_ was conducive to producing a denser microstructure than that of the control sample.

## 1. Introduction

The application of nanotechnology in construction has attracted considerable scientific interest in recent years and appears to be a promising approach towards the development of new classes of cement-based materials with superior properties [1,2,3,4]. Nanotechnology involves manipulating matter and materials in the nanometer scale below 100 nm. Nanomaterials are defined as very small particles with a size under 10^−9^ m, that are produced from the modification of atoms and molecules to produce large-scale material [5]. Most materials with a nanoscale structure were found to show positive enhancing effects on the properties of cement-based materials [6,7] owing to their fine particle size [3], high reactivity [8], and specific functional properties [5].

The existing reports in the literature review revealed that nano-SiO_2_ [9,10], nano-TiO_2_ [11], nano-CaCO_3_ [12], carbon nanotubes [13], and carbon nanofibers [14,15] are the most common nanomaterials used to modify the properties of cement-based materials. Among them, nano-SiO_2_ (NS) has received the most attention, and has been reported on intensively due to its pozzolanic reaction with portlandite, besides the filling effect. Recent experimental results have indicated that the application of nano-SiO_2_ in cementitious systems can accelerate the cement hydration rate, refine pore structure, improve strength, and increase durability [16,17,18,19,20,21,22,23,24,25,26,27,28,29,30,31,32,33,34].

In the work of Du [16], the cement hydration rate was significantly accelerated and the dormant period was significantly shortened with only a small addition of the colloidal nano-SiO_2_ due to its high surface, providing more nucleation sites and accelerating cement hydration. Compared with ordinary Portland cement, a reduction in setting time; a shortened duration of dormant and induction period of hydration; a shortening of time to reach peak of hydration; and heat evolution was also observed in nano-SiO_2_ added paste [17,18]. Moreover, during cement hydration, well distributed nano-SiO_2_ could also act as crystallization centers of hydrated products, thus increasing the hydration rate. Furthermore, as nano-SiO_2_ exhibits high pozzolanic reactivity [19,20], focus has been centered on the microstructure modification effect of nano-SiO_2_ cement-based materials [21,22,23]. It can react with calcium hydroxide to produce additional homogeneous calcium silicate hydrates (C-S-H) gel, resulting in a denser and more compact microstructure. Additionally, as nano-SiO_2_ can absorb calcium hydroxide crystals, it can reduce the size and amount of Ca(OH)_2_ crystals, making the interfacial transition zone (ITZ) of aggregates and binding paste matrix denser [24,25]. From previous experiments, the nano-SiO_2_ incorporated cement-based materials resulted in a higher compressive strength [12,21,26] and tensile strength [27,28]. With the addition of 3% nano-SiO_2_, the average compressive strength of mortar increased by 11.5%, 17.4%, 23.1%, 27.7% at 3 days, 7 days, 28 days and 90 days, respectively. From the results of the mercury intrusion porosimetry analysis, capillary pores were found to be smaller, and total porosity was also decreased by the addition of nano-SiO_2_ [21,29]. Simultaneously, the refined pore structure reduced permeability considerably (such as the calcium leaching rate and chloride ion penetration), leading to improved durability [30,31]. Ardalan et al. [32] also reported that enhanced extent of abrasion resistance of concrete samples cured in the colloidal nano-SiO_2_ environment was much greater than that of concrete samples cured in the pure water. Moreover, the enhanced extent also increased with the growth of the dosages of nano-particles. The authors also attributed this improvement to the more compact and homogeneous surface of the cement matrix caused by nano-particles.

Much work has been done to investigate the modification effects and the corresponding modification mechanism of nano-SiO_2_ on Portland cement. However, it is still unknown whether the addition of nano-SiO_2_ particles have a similar effect on AC$A cement, despite the study conducted by Ma [35] on the influence of nano-TiO_2_ on the sulphoaluminate cement hydration process.

AC$A cement, also called high calcium sulphoaluminate cement, performs well as an energy-saving cement [36]. AC$A cement clinker containing sulphoaluminate (C_4_A_3_$) can be synthesized through annealing [37,38], or by mineralizers such as CaF_2_ [39,40], CuO [41] and ZnO [42]. It is prepared by introducing the mineral C_4_A_3_$ into ordinary Portland cement clinker. When compared with Portland cement, AC$A cement has a quick setting time, high early strength, and other excellent characteristics such as good anti-freezing and lower alkalinity [37].

To further research this topic, the objective of this investigation was to identify the effects of nano-SiO_2_ on the early hydration of AC$A cement. The properties of AC$A cement pastes containing different dosages of nano-SiO_2_ were studied per the determination of hydration kinetics, hydration products, porosity and pore size distribution, and microstructure. Corresponding analytical techniques were conducted using isothermal calorimetry, X-ray diffraction (XRD), thermogravimetric/derivative thermogravimetric (TG/DTG), mercury intrusion porosimetry (MIP) and scanning electron microscopy (SEM), respectively. These results may help provide a comprehensive explanation and a practical guide for the nano-modification effects of nanomaterials on cement-based materials.

## 2. Materials and Methods

### 2.1. Materials

**Figure 1:**
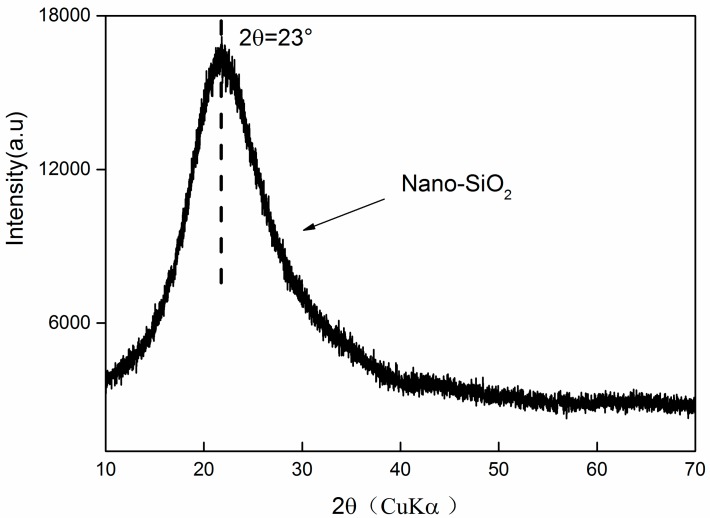
X-ray diffraction (XRD) spectra of nano-SiO_2_.

**Figure 2:**
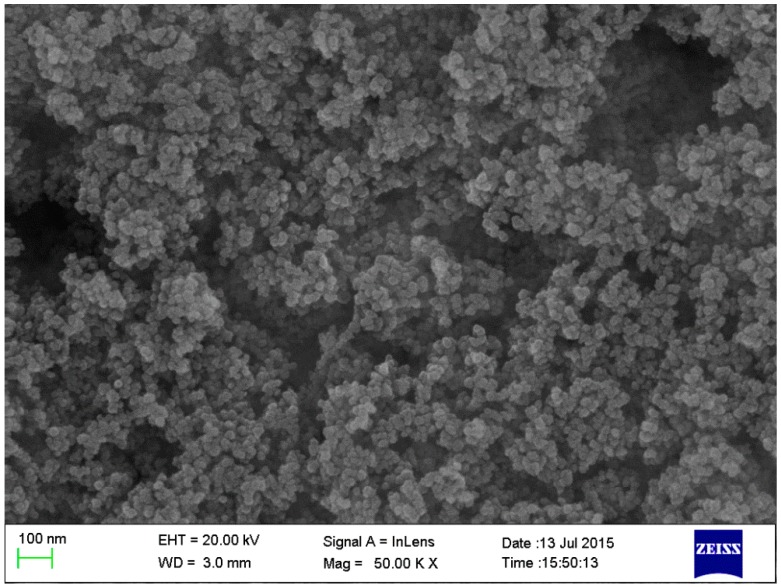
Field emission scanning electron microscopy (FESEM) micrograph of nano-SiO_2_ powder (magnification = 50,000).

AC$A clinker was synthesized using industrial raw materials in a laboratory according to the method proposed in Reference [38]. Next, the resulting products were homogenized with CaSO_4_·2H_2_O in a mass ratio of 4% to produce AC$A cement. The homogenization of the samples was performed by ball milling for 12 h. The Blaine surface area, average particle size, and density of the AC$A cement were 350 m^2^/kg, 23.8 μm, and 3.2 g/cm^3^, respectively. The chemical compositions of the materials (Table 1) were determined through X-ray fluorescence (XRF, ARL ADVANT’XP, Thermo Fisher Scientific, Waltham, MA, USA), and the mineralogical composition of the un-hydrated cement was quantified by Rietveld analysis. Nano-SiO_2_ was supplied by Aladdin Industrial Corporation from Shanghai, China. The main properties of the nano-SiO_2_ particles are summarized in Table 2. The crystal structure and morphology of nano-SiO_2_ were investigated by XRD and field emission scanning electron microscopy. From the XRD pattern (Figure 1), the characteristic diffraction broad peak centered on 23° (2*θ*) confirmed its amorphous nature. The morphologic graph shown in Figure 2 indicates that nano-SiO_2_ is spherical in shape, but does not disperse well. Furthermore, it can be clearly seen that the average particle size of the nano-SiO_2_ was approximately 15–20 nm, which agrees with the data in Table 2.

### 2.2. Methods

#### 2.2.1. Specimen Preparation

To detect the effects of nano-SiO_2_ on the properties of the AC$A cement paste, we replaced 1% and 3% of the cement by weight with a nano-SiO_2_ addition when preparing the paste specimens, as seen in Table 3. For all experiments, an effective water-to-solid ratio of one was maintained. As aggregations occurred between the nano-SiO_2_ particles (see Figure 2), dispersion measures were taken to reduce the presence of matrix defects in the paste specimens. To achieve an equal dispersion of nano-SiO_2_, the nano-SiO_2_ particles were first dispersed in water via a high intensity ultrasonic bath for 2 min. Next, the dispersed nano-SiO_2_ was mixed with the cement in the mixing machine and stirred for 5 min. Finally, the resulting paste was put into a hollow cylindrical plastic mold and stored at 20 ± 1 °C and 95% relative humidity (RH). The paste hydration was stopped by solvent exchange using ethanol until they were utilized for experimental characterization. At each specified age, samples were immersed in ethanol for 24 h to stop the hydration.

#### 2.2.2. Determination of Heat of Hydration

An 8-channel isothermal calorimeter (TAM Air; Thermometric AB, Jarfalla, Sweden) was used to determine the heat evolution during hydration. To ensure accurate early age measurement and minimize the time for isothermal conditions, the nano-SiO_2_ particles and water were conditioned in a curing box for 24 h at 20 °C. Next, they were mixed using a mixing machine for 2 min. Approximately 10 g of sample was immediately extracted, placed in a sealed sample vial to minimize evaporation, and placed in the isothermal calorimeter to determine the heat release response. Heat flow curves were recorded for 72 h under a constant temperature of 20 °C.

#### 2.2.3. Determination of Phase Development

XRD was used to determine the mineralogical composition of the basic materials and the evolution of the hydrated phase at different curing ages. The XRD patterns of the pastes were tested on a Rigaku SmartLab 3000A diffractometer (Tokyo, Japan), with Cu Kα radiation (wavelength = 0.154 nm). The accelerate voltage, accelerate current, step size of the tests were 40 kV, 20 mA, and 0.05, respectively. After the samples in Section 2.2.2 were removed from the ethanol, they were first pulverized in ethanol with an agate mortar and passed through an 80 μm sieve as vacuum filtrating. The powder was dried at 40 °C for 12 h in a vacuum drying oven. Data was collected in a scanning range of 5–60°, with a speed of 5° per minute.

#### 2.2.4. Determination of Calcium Hydroxide Content

**Figure 3:**
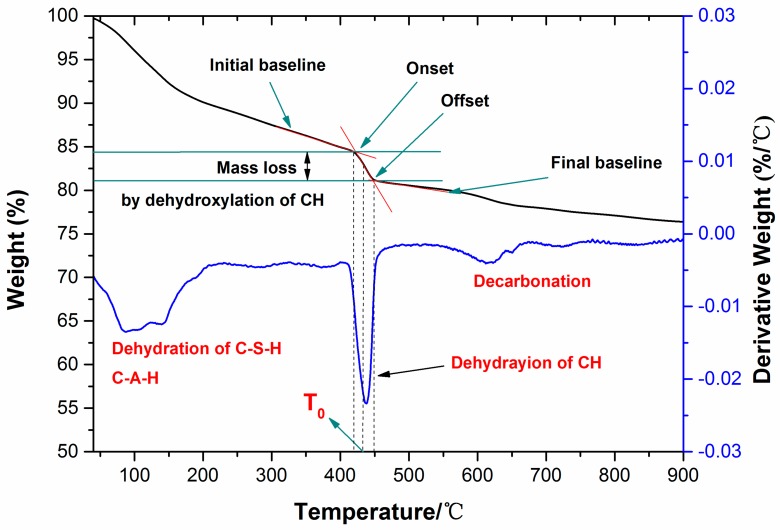
Method used to calculate the calcium hydroxide content of paste.

Thermal gravimetric analysis (TGA/DSC, NETZSCH, ATA409, NETZSCH, Selb, Upper Franconia, Germany) has been widely used to identify hydration products, including ettringite, monosulphate (AFm), and calcium hydroxide (CH), and has been accepted as an accurate method for the determination of crystalline calcium hydroxide content [43,44]. The samples for thermal gravimetric analysis were prepared in a similar method to the samples used for XRD analysis. TG-DTG analysis was conducted using a simultaneous thermal analyzer with a uniform heating rate of 10 °C per minute from 40 to 1000 °C under nitrogen flow. The weight loss of the samples was calculated and the reactions occurring with an increase of temperature in the samples are discussed in References [45,46]. The calcium hydroxide content was determined using the method described by a graphical technique used previously in References [43,44] and is illustrated in Figure 3.

Within the TG curve, the onset and offset points of the decomposition curve of CH were determined to be the intersections of two tangent lines. These were defined as the initial baseline and final baseline, respectively. Using the onset and offset points (temperature axis), the mid-point T0 was created. The weight loss mH_2_O was defined as the distance between two intersections generated by the vertical line from T0 with the initial and final baselines. Next, the content of CH (m_CH_) was calculated by the following equation:

(1) $$Ca{\left(\mathrm{OH}\right)}_{2}\to CaO+H_{2}O$$

(2) $$m_{CH}=m_{H_{2}O}\times \frac{74}{18}$$

where 74, and 18 are the molar weight of Ca(OH)_2_ and H_2_O, respectively.

#### 2.2.5. Determination of Porosity and Pore Size Distribution

The mercury intrusion porosimetry (MIP) technique was used to quantitatively evaluate the pore structures of cementitious materials to provide a valid measurement of total porosity. This technique was based on the capillary law expressed by the modified Washburn equation: *d* = −2γcos*θ*/P, where d is the pore entry radius in which mercury is introduced; γ is the surface tension; *θ* is the contact angle; and P is the applied pressure. The measurement was performed with a Quanta chrome PoreMaster GT60 (Quantachrome, Houston, TX, USA) mercury intrusion porosimeter with a high pressure range of 140–420 kPa, and a low pressure range of 1.5–350 kPa. Pore sizes ranging from 0.0035 to 400 μm were recorded. Prior to testing, bulk samples with a diameter from 3 to 6 mm were taken out of the ethanol for immersion for 24 h, dried at 40 °C for 12 h in a vacuum drying oven.

#### 2.2.6. Determination of Microstructure and Morphology

Scanning electron microscopy (FESEM, HITACHI S4800, Hitachi Limited, Tokyo, Japan) was used to observe microstructure and morphology changes in the hydration products. Measurements were performed with a JSM-5900 SEM operated at 30 kV. A few pieces of sample material were taken from the paste specimen and immersed into ethanol to terminate hydration. Before testing, the samples were removed from the ethanol, and dried at 40 °C for 7 days in a vacuum drying oven. The surface of the samples was sputtered with gold.

## 3. Results and Discussion

### 3.1. Isothermal Calorimetric Analysis

Many properties of Portland cement, such as strength development, setting and hardening, are associated with the hydration process. Thus, the influence of nano-SiO_2_ on hydration kinetics will first be discussed in this section.

**Figure 4:**
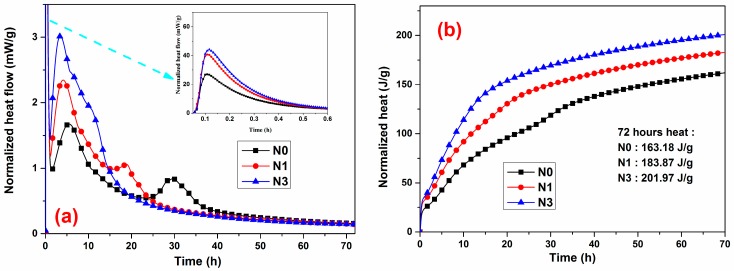
(**a**) Heat flow curves and (**b**) cumulative heat curves for AC$A cement. Inset is the magnified part in the time range from 0 to 0.6 h.

The heat evolution curves describing the rate of heat evolution for the cement/nano-SiO_2_ mixtures with 0%, 1% and 3% addition of nano-SiO_2_ are presented in Figure 4. Like typical Portland cement [47,48], the hydration process of AC$A cement can also be divided into five stages: the initial reaction period; the induction period; the acceleration period; deceleration period; and the slow reaction period. The initial peak in the first stage (from left to right), can be attributed to a combination of exothermic wetting and early-stage reactions, including the superficial reaction of C_3_S, the rapid dissolution of free lime and aluminate phases, and the immediate formation of ettringite. The main peak corresponded to the end of the acceleration period, where the main products, as with Portland cement, were C–S–H and CH. An affiliated peak right after the main hydration peak was associated with the renewed dissolution of tricalcium aluminate (C_3_A) and the formation of ettringite [49].

As Figure 4 illustrates, the addition of nano-SiO_2_ did not remove or add additional peaks, but changed the intensities or time they occurred. When compared with the reference sample, the main peak occurred earlier as the nano-SiO_2_ dosage increased, indicating that nano-SiO_2_ increased the hydration rate of C_3_S. Obviously, the formation of AFm could have been obtained at an earlier stage when nano-SiO_2_ was added to the paste, and a possible reason was examined in Reference [50]. The addition of nano-SiO_2_ powders significantly increased the intensity of the heat peak and shortened its duration of occurrence. The increase in nano-SiO_2_ dosage from 1% to 3% enhanced these effects, but did not trigger a proportional increase in the heat release rate. Figure 4 shows the curves of the total heat evolution during the first 72 h of hydration. This corresponds to the trend of heat evolution rate as the incorporation of the nano-SiO_2_ powders resulted in a greater cumulative heat release. Such a great heat release was mainly due to the presence of nano-SiO_2_ accelerating the reaction process in the cement based system. Said [21] suggested that the acceleration effect of nano-SiO_2_ on the hydration of cement-based materials was related to its considerably fine particle size with high surface area, which could act as potential heterogeneous nucleation sites for the hydration products to deposit, thus increasing the hydration rate and decreasing the induction period. Moreover, nano-SiO_2_ exhibited ideal pozzolanic activity owing to its amorphous nature [19,20], which led to the formation of additional C–S–H. The additional C–S–H acted as additional nucleation sites [9], promoting the formation and growth of reaction products from the dissolved units. Consequently, the reaction process was accelerated.

Thus, the results obtained in isothermal calorimetry showed that nano-SiO_2_ was an active additive that significantly accelerated the hydration kinetics of AC$A cement, thus increasing the amount of heat released.

### 3.2. X-ray Diffraction Analysis

**Figure 5:**
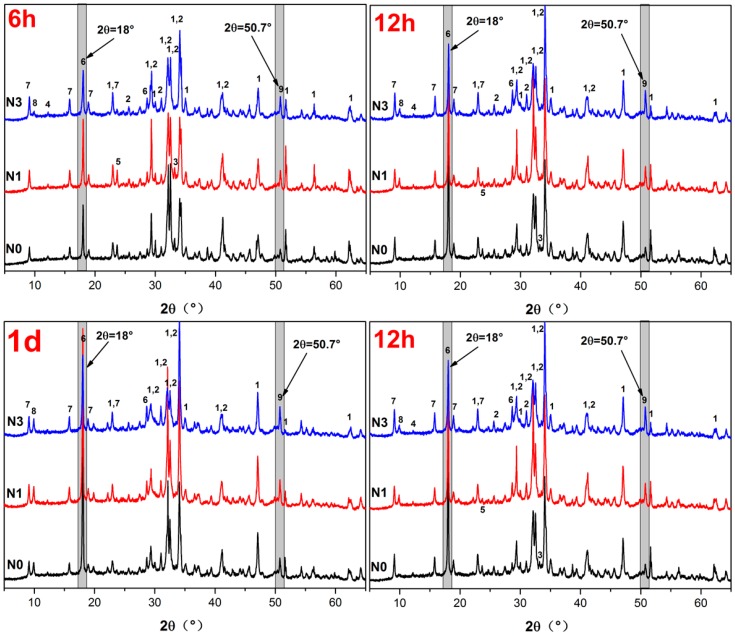
XRD patterns of samples hydrated for different curing ages (1-alite, 2-belite, 3-tricalcium aluminate, 4-brownmillerite, 5-sulphoaluminate, 6-CH, 7-ettringite, 8-AFm, 9-crystalline C–S–H).

As nano-SiO_2_ was observed to have a significant acceleration effect on the hydration kinetics of AC$A cement (as illustrated in Figure 5), the effect on phase development was further investigated through detailed XRD studies. Each peak shift and intensity was monitored and carefully analyzed. For comparison, the peaks of CH (2*θ* = 18°) and crystalline C–S–H (2*θ* = 50.7°, jennite and tobermorite) [51] were first selected (see grey area in Figure 5). In Figure 5, a peak in CH was observed in the N0 mix representing the hydration product (CH), which was released during the hydration of the AC$A cement. At 6 h, the intensity of the CH peak evidently increased with the addition of nano-SiO_2_, especially at 1% content, which reflected the accelerated cement hydration reaction by nano-SiO_2_ during this period. However, the intensity of the CH peak decreased with the addition of nano-SiO_2_, and was significantly reduced with nano-SiO_2_ when time was increased from 12 h to 3 days, which can be mainly attributed to the high pozzolanic reactivity of the nano-SiO_2_ consuming the CH in the paste at a later stage. In contrast, during all time periods, the intensity of the C–S–H peak remarkably increased in each mix when compared to the control mix. This was due to the nucleating effect of nano-SiO_2_, which accelerated the growth of the hydrated products and the pozzolanic effect with CH which formed an additional C–S–H as per Equations (3)–(5) [51]. In the pastes with added nano-SiO_2_, AFm formed at the very early stages of hydration (within 6 h), and its intensity increased with nano-SiO_2_, whereas there was no AFm present in the control specimen. These results cohere well with the results of hydration heat in Section 3.1.

(3) $$nano-SiO_{2}+H_{2}O\to H_{2}SiO_{4}^{2-}$$

(4) $$Ca{\left(OH\right)}_{2}+H_{2}O\to Ca^{2+}+OH^{-}$$

(5) $$H_{2}SiO_{4}^{2-}+Ca^{2+}\to C-S-H\left(additional\right)$$

As shown by the calorimetric analysis in Figure 4, the hydration process accelerated significantly with the increase in nano-SiO_2_. Therefore, the intensities of the X-ray diffraction peaks (Figure 5), corresponded to the unreacted cement minerals with the nano-SiO_2_ addition lower than those of the control specimen.

Apart from these major characteristic features of the diffraction patterns, it was observed that the main hydrated products of both the control sample and the sample containing nano-SiO_2_ shown in Figure 5 were the same: portlandite, ettringite, AFm, and C–S–H were the major hydration products for all samples, implying that nano-SiO_2_ had no influence on the hydrated products [52].

### 3.3. TG/DTG Analysis

**Figure 6:**
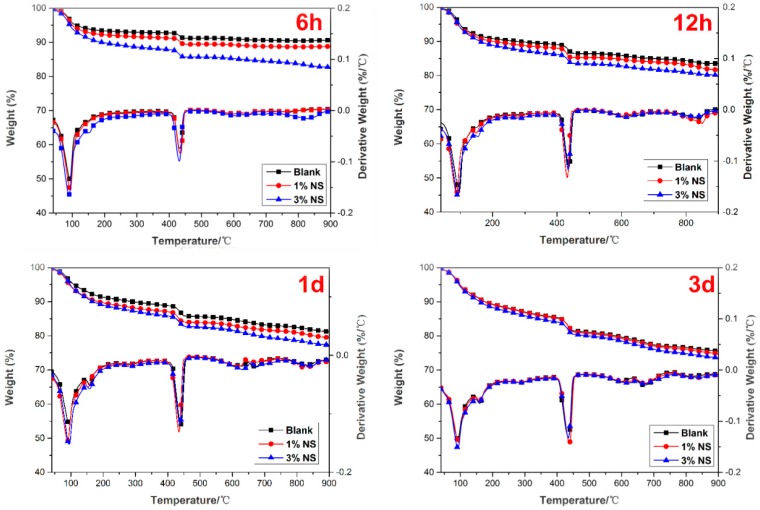
Results of the thermogravimetric analysis (TGA).

The results of the TG/DTG analysis for the samples containing nano-SiO_2_ hydrated for different curing ages are illustrated in Figure 6; curves are represented as functions of furnace temperature. The trend of mass loss was similar, but the differences are rather visible among these TG curves. It can be clearly seen that the overall mass loss of the samples changed with the amount of nano-SiO_2_ and curing time (see Table 4). It was observed that the weight loss of the control paste increased from 9.18% to 25.51% with an increase from 6 h to 3 days. As for the paste with the 1% nano-SiO_2_ addition, the weight loss increased from 11.06% to 26.18% with the testing period from 6 h to 3 days. When comparing the weight loss of all paste samples at 3 days, the 3% NS addition shows the highest value, which is 27.36%. The overall mass loss can be used to explain the acceleration effect of nano-SiO_2_ directly on AC$A cement hydration.

From the DTG curve, it was possible to determine the beginning and end of each step which was represented by the change in the slope of the TG curve. Based on this result, three major weight loss processes could be observed during heating of the samples. The first weight loss between approximately 40 °C and 200 °C was attributed to the removal of the absorbed water and dehydration reactions of several hydrated products such as C–S–H, ettringite, monosulfoaluminate and gehlenite. The dehydration of the C–S–H was the major cause of mass loss. The second mass loss corresponding to the pitch in the temperature range 380–460 °C in Portland cement pastes was due to the decomposition of Ca(OH)_2_ as per the reaction. A broad and smooth peak occurred during the third weight loss process at 600–1000 °C, representing the decarbonation of the different forms of calcite. Moreover, surface area, particle size, impurities were all factors that affected the decomposition of calcium carbonate.

The content of CH in the pastes determined by TGA and the total mass losses during the heating process are presented in Table 5. Compared with the control sample, a higher amount of CH (5.29% for control sample, 6.19% for 1% NS, and 2.41–3.57% for 3% NS) was observed in the nano-SiO_2_ incorporated AC$A cement system up to 6 h. This was attributed to the hydrated products of cement particles deposited on the surface of nano-SiO_2_, which acted as nucleation sites and accelerated AC$A cement hydration during this period. However, at a later stage, i.e., from 12 h to 3 days, the amount of CH was lower than that of the control sample, especially the 3% nano-SiO_2_ at 3 days, indicating that consumption of CH in the pozzolanic reaction was more dominant than the nucleation effect of nano-SiO_2_ itself. This was also consistent with a previous study in Reference [53]. The results obtained from TG/DTG analysis were further confirmed by the XRD results.

### 3.4. Pore Structure Analysis

MIP has been widely used in the quantitative characterization of pore structure in cement-based materials where a wide range of pore sizes from 0.001 μm to 1000 μm can be measured [54,55]. Therefore, both the porosity and the pore size distribution of cement paste can be obtained through this technique. According to previous research [12], the pores in hydrated cement paste are classified into three parts: 10–0.05 μm, 0.05–0.01 μm and <0.01 μm, which represents large capillary pores, medium capillary pores and gel pores, respectively.

**Figure 7:**
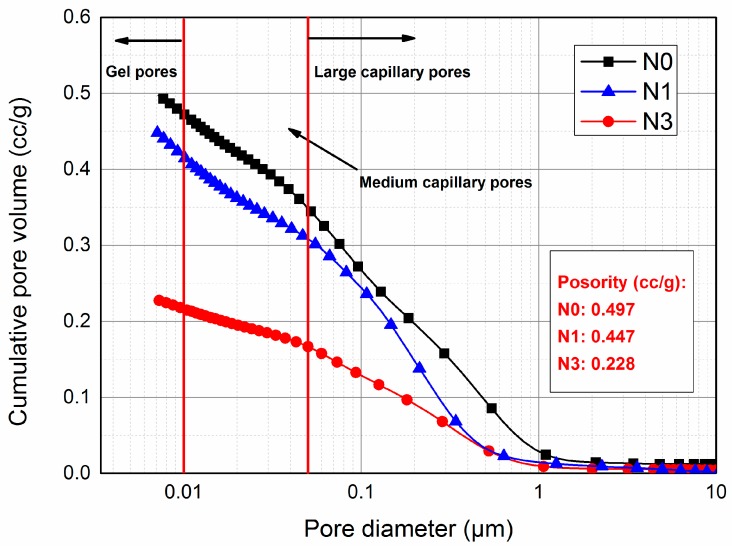
Cumulative pore volume of NS blended AC$A cement pastes hydrated at 3 days.

**Figure 8:**
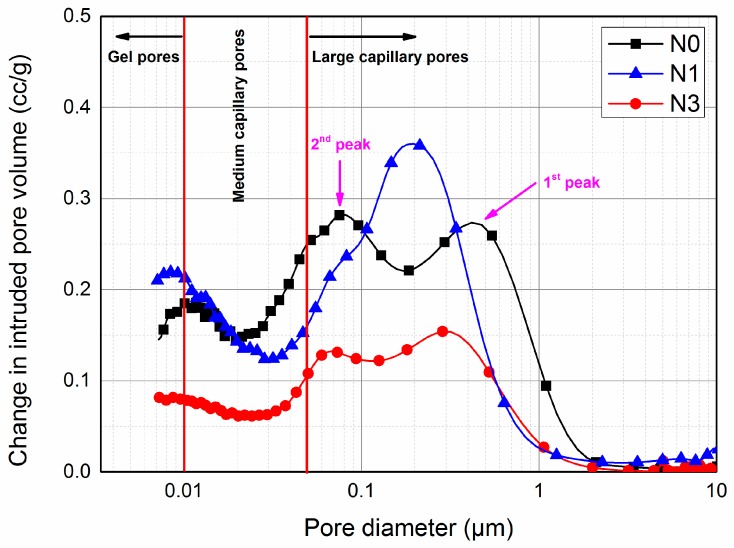
Pore size distribution of NS blended AC$A cement pastes hydrated at 3 days.

The porosity analysis of blended AC$A cement pastes using mercury intrusion porosimetry after 3 days curing is shown in Figure 7 and Figure 8. The results indicated that nano-SiO_2_ had an important effect on the pore structure of hardened AC$A cement paste. It was observed that the cumulative pore volumes of these pastes (Figure 7) decreased significantly with the addition of nano-SiO_2_ into the AC$A cement. The decrease in the apparent total porosities for mixtures N1 (1% nano-SiO_2_) and N3 (3% nano-SiO_2_) was 9.91% and 54.09%, respectively, relative to that of mixture N0 (49.75 cc/g).

The pore size distribution (the variation of dV/d log V with pore diameter) of blended AC$A cement pastes are plotted in Figure 8. The region under the curve represents the concentration of the pores and the value of the critical diameter corresponded to the peak value of the log differential curve from the MIP results [12]. Based on the curves, it was clear that the addition of nano-SiO_2_ had a significant effect on the pore size distribution. The first peak value (maximum concentration of pores) in N0 was 0.45 μm, whereas the peak values shifted to smaller regions in N1 and N3, corresponding to 0.2 μm and 0.31 μm, respectively. However, the difference among all three samples was not obvious in terms of the second and third peak values. As seen in Table 5, with 1% nano-SiO_2_ addition, capillary pores (including large and medium capillary pores) with a diameter between 0.01 and 10 μm became smaller than the control sample, while the gel pores became larger. However, for the 3% nano-SiO_2_ addition, the volume of both capillary and gel pores decreased significantly at 53.90% and 51.81% less than N0, respectively. These results were also consistent with the curves shown in Figure 8.

The above-mentioned results demonstrate that the presence of nano-SiO_2_ is advantageous for the pore structure refinement of AC$A cement pastes. These positive effects are ascribed to the filling effect of nano-SiO_2_, the promoted formation of C–S–H via the nucleation effect, and the pozzolanic reaction between nano-SiO_2_ and CH. Since MIP only detects interconnected pores, the capillary pores were blocked by nano-SiO_2_ (which act as fillers) and hydration products (including additional C–S–H), resulting in the decreased interconnection of these pores and improved pore structure [56]. However, the contribution of each effect in refining pore structure remains difficult to differentiate.

### 3.5. Microstructure Analysis

**Figure 9:**
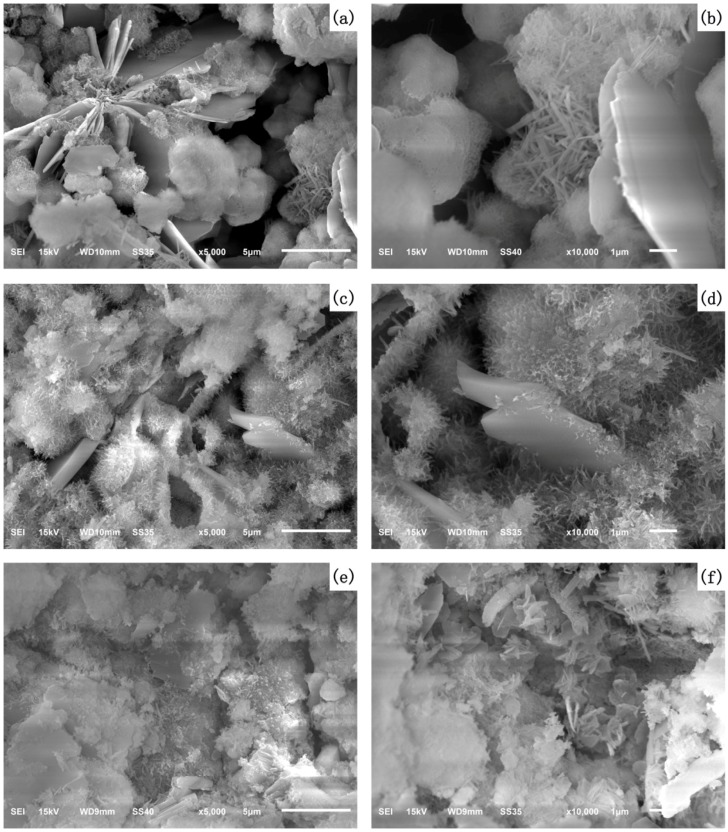
Scanning electron microscope (SEM) micrographs of (**a**,**b**) N0 specimens; (**c**,**d**) N1 specimens; and (**e**,**f**) N3 specimens at 3 days.

The influence of nano-SiO_2_ on the microstructure of the AC$A cement paste cured up to 3 days is shown in Figure 9. As seen in Figure 9a,b, portlandite, C–S–H gels, pores and unhydrated AC$A cement particles were the main constituents of the AC$A cement paste, and short rods of ettringite can also be seen in the hydration product. When nano-SiO_2_ was added, the C–S–H gels became relatively dense and compact, as shown in Figure 9c–f. As seen clearly in the SEM micrographs, the microstructure of the hardened AC$A cement paste was found to have fewer voids with the increase in nano-SiO_2_ content in the specimens. Furthermore, the formation of smaller portlandite crystals was also found for 1% and 3% at 3 days compared to 0% nano-SiO_2_ content specimens, which was consistent with the results obtained by Kong et al. [51]. These micrographs provide visual evidence of the microstructural characteristics responsible for the decrease in porosity. Additionally, the SEM analysis revealed that nano-SiO_2_ led to a great improvement in microstructure. These improvements can be explained as follows: first, the high surface energy of the nano-SiO_2_ particles were uniformly dispersed in the AC$A cement paste, providing a nucleus for the growth of hydration products in their vicinity. Second, the nano-SiO_2_ particles filled the voids and reacted with the cement hydration products to form additional C–S–H, which compacted the microstructure of the paste.

## 4. Conclusions and Recommendations

Tests were conducted to investigate the effects of nano-SiO_2_ on AC$A cement hydration. Based on the limited experimental study, corresponding conclusions can be summarized as follows:

1. The incorporation of nano-SiO_2_ significantly accelerated the hydration process kinetics of AC$A cement, promoting C_3_S hydration which resulted in a greater cumulative heat release.
2. Portlandite, ettringite, AFm, and C–S–H were found to be the major hydration products for all samples, indicating that nano-SiO_2_ had no effect on the kind of hydration product.
3. The addition of nano-SiO_2_ led to a significant increase in CH content due to nucleation effect up to 6 h. However, since pozzolanic reactions are more dominant than the nucleation effects of nano-SiO_2_, an increased consumption of CH from 12 h to 3 days was more obvious in the nano-SiO_2_ incorporation pastes.
4. The inclusion of nano-SiO_2_ led to a great improvement in microstructure. The total porosity and capillary pores decreased with increasing nano-SiO_2_ content. More refinement of the pore structure was achieved by increasing the nano-SiO_2_ content up to 3%.

To summarize, the addition of nano-SiO_2_ can significantly accelerate early hydration, optimize pore structure, and the microstructure of AC$A cement. Nevertheless, further research is required into the effects of nano-SiO_2_ on the mechanical and long-aged hydration properties of AC$A cement.

## Figures and Tables

**Table 1:** Properties of cement.

Oxide Content		Mineralogical Composition	
Oxide	(% of Mass)	Phase	(% of Mass)
Calcium oxide, CaO	63.9	Tricalcium silicate, C_3_S	48.16
Silicon dioxide, SiO_2_	19.8	Dicalcium silicate, C_2_S	31.75
Aluminium oxide, Al_2_O_3_	4.4	Tricalcium aluminate, C_3_A	5.70
Sulphur trioxide, SO_3_	3.8	Ferrite, C_4_AF	4.61
Ferric oxide, Fe_2_O_3_	3.1	Free-lime, f-CaO	1.48
Magnesium oxide, MgO	1.6	Magnesium oxide, MgO	1.35
Potassium oxide, K_2_O	0.4	Calcium sulphoaluminate, C_4_A_3_S	5.23
Sodium oxide, Na_2_O	0.1	Gypsum, CS	1.61
Loss on ignition	2.2		

**Table 2:** Properties of nano-SiO_2_.

Color	Diameter (nm)	Crystal Type	Surface Volume Ratio (m^2^/g)	Purity (%)	pH Value
White	15 ± 5	amorphous	63	99.5	6

**Table 3:** Mixes investigated.

Mix Designation	AC$A Cement (%)	Nano Silica (%)
N0	100	0
N1	99	1
N3	97	3

**Table 4:** TGA analysis of the weight loss of pastes at the ages of 6 h, 12 h, 1 d, and 3 d.

	Mass Loss (%)		
Mix	Evaporable Water + Nonevaporable Water (C–S–H + Ettringite + Monosulfoaluminate)	Portlandite from TG (%)	Overall Weight Loss (%)
Control-6 h	7.04	5.29	9.18
Control-12 h	10.20	8.64	16.39
Control-1 d	10.12	9.75	19.64
Control-3 d	13.00	11.15	25.51
1% NS-6 h	8.37	6.19	11.06
1% NS-12 h	11.23	8.31	18.72
1% NS-1 d	11.88	9.53	21.64
1% NS-3 d	13.13	11.08	26.18
3% NS-6 h	11.38	6.41	17.22
3% NS-12 h	12.70	8.43	19.79
3% NS-1 d	12.82	9.58	23.42
3% NS-3 d	14.08	10.55	27.36

**Table 5:** Mercury intrusion porosimetry (MIP) test results.

Mixture	Apparent Total Porosity (cc/g)	Volume of Large Capillary Pores (cc/g)	Volume of Medium Capillary Pores (cc/g)	Volume of Gel Pores (cc/g)
N0	49.75	33.6	12.39	2.49
N1	44.82	30.64	10.80	3.37
N3	22.84	16.27	4.93	1.20
